# Hydrogen and Methane Detection in Breath in Response to Two Different Types of Dietary Fiber and Its Relationship to Postprandial Glucose Concentration in Obese Patients with Type 2 Diabetes and Normoglycemic Subjects

**DOI:** 10.3390/nu17050917

**Published:** 2025-03-06

**Authors:** Inna Misnikova, Yulia Kovaleva, Svetlana Shokur, Tyler W. LeBaron, Oxana Povarova, Oleg Medvedev

**Affiliations:** 1 M.F. Vladimirski Moscow Regional Research and Clinical Institute, Schepkina 61/2, 129110 Moscow, Russia; inna-misnikova@mail.ru (I.M.); yulia.kovaleva@mail.ru (Y.K.);; 2 Department of Kinesiology and Outdoor Recreation, Southern Utah University, Cedar City, UT 84720, USA; tylerlebaron@suu.edu; 3 Molecular Hydrogen Institute, Enoch, UT 84721, USA; 4 Department of Pharmacology, M. V Lomonosov Moscow State University, Lomonosovsky Prospect 27-1, 119991 Moscow, Russia; medvedev@fbm.msu.ru; 5 National Medical Research Center of Cardiology, Laboratory of Experimental Pharmacology, Academician Chazov Str., 15a, 121552 Moscow, Russia

**Keywords:** dietary fiber, molecular hydrogen, methane, type 2 diabetes, postprandial glucose levels

## Abstract

**Background:** The aim of this study was to investigate the relationship between postprandial glycemic levels based on flashmonitoring and the production of intestinal hydrogen (H_2_) and methane (CH_4_) gases based on the measurement of the amount of these gases in exhaled air. **Materials and Methods**: We studied 14 subjects with type 2 diabetes mellitus (T2DM) and 14 individuals without diabetes (control) with two food load tests, including two types of dietary fiber (inulin and guar gum), with the simultaneous determination of gases in exhaled air and the assessment of glucose levels. **Results**: All subjects in the control group had a significant increase in exhaled H_2_. OR for increased hydrogen production in patients with T2DM was 0.17 (95% CI 0.031–0.93, *p* = 0.043). The level of H_2_ in exhaled breath after food load in patients with T2DM was lower than in normoglycemic subjects. There was an inverse correlation between maximum glucose rise and maximum H_2_ in exhaled air after food load in normoglycemic subjects (r = −0.569, *p* = 0.034). Patients with T2DM had direct correlations between the level of CH_4_ in exhaled air and the parameters of postprandial glycemia in the lactulose test (*p* < 0.05). **Conclusions:** The confirmation of a causal relationship between decreased H_2_ production, increased intestinal CH_4_ production, and more severe postprandial glycemia may identify new therapeutic targets in the correction of postprandial glycemia in patients with T2DM.

## 1. Introduction

The dietary approach of patients with type 2 diabetes mellitus (T2DM) is recognized as an important task for maintaining optimal glycemic control and reducing the risk of late complications. Dietary fiber (DF) is now an essential component of a healthy diet. According to World Health Organization(WHO) recommendations, the daily dose of DF for adults should be at least 25 g [1]. According to the Academy of Nutrition and Dietetics (USA), the amount of DF per day should be equal to 14 g of fiber per 1000 kcal, which generally corresponds to approximately 25 g/day for adult women and 38 g/day for adult men [2]. For patients with T2DM, it is also important to include DF in amounts equivalent to those without T2DM [3]. Recommendations to increase the amount of DF in the diet are driven by the extensive evidence that DF reduces the risk of a range of chronic diseases. For example, DF intake is associated with the reduced risk of cardiovascular disease [4], colorectal cancer [5,6], T2DM [7], and obesity [8]. Fiber intake is associated with a reduction in total cholesterol and low-density lipoprotein cholesterol (LDL-C).

DF affects the absorption of carbohydrates [9] and contributes to maintaining a healthy intestinal microbiota metabolism by serving as a substrate for its fermentation. This fermentation process yields short-chain fatty acids (SCFAs) [10].In addition to the formation of SCFAs, the anaerobic fermentation of carbohydrates by the intestinal microbiota initially produces hydrogen (H_2_) and, in some humans, methane (CH_4_) [11]. These gases are partially absorbed through the intestinal wall into the blood and eliminated from the body by respiration. Therefore, an increase in the concentration of intestinal gases in exhaled air indicates the beginning of the fermentation process of DF by gut bacteria [12,13,14]. This principle underpins the basis of the respiratory test for the detection of small intestinal bacterial overgrowth (SIBO) syndrome [15]. SIBO is characterized by the presence of at least >105 colony-forming units of colonic bacteria per mL of jejunal aspirate [16]. In general, the colonic bacteria in humans produce between 35 and 321 mL of H_2_ per day [17], and perhaps as high as 12 L/day [18]. Thus, methane-producing archaea can metabolize H_2_ and carbon dioxide (CO_2_) [19], resulting in the production of CH_4_, which can also be determined via breath tests [20]. Therefore, the composition of the intestinal microbiota, in particular the presence of glycolytic bacteria and methane-producing archaea, can be indirectly estimated from breath tests.

H_2_ has recently been shown to have antioxidant, anti-inflammatory, and antiapoptotic effects [21,22,23]. H_2_ molecules freely diffuse into cells and neutralize the most reactive oxygen species (ROS), such as hydroxyl radicals and peroxynitrite, which in turn reduces the production of proinflammatory molecules and subsequent apoptosis. In contrast, there is little evidence that methanogenic bacteria provide therapeutic effects, and may even be implicated in various adverse effects [24,25,26].

The production of H_2_ and CH_4_ depends on the amount and type of DF, which is a heterogeneous group of compounds with different physicochemical properties [27]. This may account for differences in their effects on metabolism in both patients with T2DM and normoglycemic subjects. In addition, these differences may be due to phenotypic, genetic, and other characteristics specific to individuals. Currently, along with the recommendation to increase the intake of whole grains, vegetables, and fruits as a source of DF, DFs that are produced from natural raw materials are being incorporated into healthy diets to provide more fiber in a small quantity [28]. These include inulin and guar gum [29,30,31]. Their addition makes it possible to saturate the diet with DF and achieve the recommended daily intake of DF. The main reasons for choosing food fibers such as inulin and guar gum in our study are their different chemical compositions and physicochemical characteristics. Guar gum belongs to the soluble, viscous fibers. Chemically, guar gum is an exo-polysaccharide composed of the sugars galactose and mannose. Inulin is a heterogeneous collection of fructose polymers that belongs to the soluble, non-viscous fibers. These two food fibers differ in their effects on glycemia control [32].

In the literature available to us, only two studies examined the effect of DF on the level of postprandial glycemia with an assessment of the state of the intestinal microflora. These studies examined the effect of chronic dietary fiber intake on microbiome health and changes in glucose levels [33,34]. Only one of these studies was conducted with subjects at cardiometabolic risk [34]. The association between the level of intestinal gases (H_2_ and CH_4_) in exhaled air with postprandial glycemia as measured by flash glucose monitoring in patients with T2DM and normoglycemia has not been studied. Therefore, the aim of our study was to evaluate the level of CH_4_ and H_2_ in breath in response to two different types of dietary fiber (inulin and guar gum) and its relationship to postprandial glucose concentration in obese patients with type 2 diabetes and normoglycemic subjects.

## 2. Materials and Methods

### 2.1. Study Design and Subjects

Fourteen patients with T2DM and fourteen individuals without diabetes (control group) were included in the study. The study included Caucasian patients with T2DM living in the Moscow region and referred for planned hospitalization to the Department of Therapeutic Endocrinology of M.F. Vladimirski Moscow Regional Research and Clinical Institute. The control group consisted of healthy Caucasian volunteers. The study was approved by the Local Ethic Committee (protocol #5 of 14 April 2022).

Inclusion criteria for patients with T2DM were as follows: established diagnosis of T2DM; body mass index (BMI) > 30 kg/m^2^; age between 40 and 70 years; acceptable drugs, such as those from the groups of sodium-glucose co-transporter 2 inhibitors (SGLT-2i), dipeptidyl peptidase-4 inhibitors (DPP-4i), metformin, sulphonylurea (SU), and insulin; willingness and ability to perform all study procedures; and signed informed consent.

Exclusion criteria for patients with T2DM were as follows: T1DM; use of short- or ultra-short-acting insulin; use of oral sugar-lowering drugs except metformin, SU, SGLT-2i, and DPP-4i; and gastrointestinal form of autonomic neuropathy.

Inclusion criteria for the control group were age 25–55 years and signed informed consent. Exclusion criteria for the control group were the presence of T1DM, T2DM, obesity, or metabolic syndrome.

General exclusion criteria for all study subjects were as follows: inflammatory bowel disease; taking antibiotics within 1 month before the study; taking pre- and probiotics within 1 week before the study; taking prokinetics and laxatives, including lactulose, within 1 week before the study; having undergone colonoscopy or irrigoscopy within the last 4 weeks before the study; and impaired cognitive abilities.

### 2.2. Supplementation Protocol

**Figure 1:**
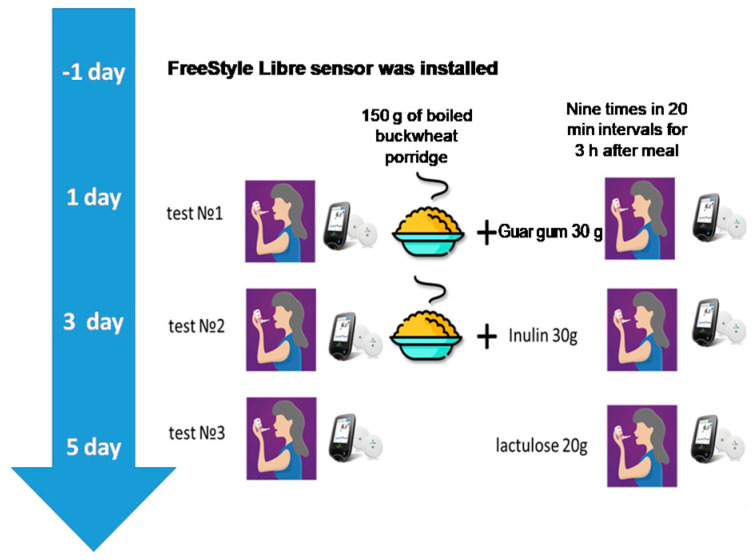
The study design and time frame of the study.

Twenty-four hours before inclusion in the study, subjects were advised to exclude foods containing DF from their diet. During the study, subjects underwent 2 food load tests with simultaneous determination of gases in exhaled air (performance of hydrogen–methane breath test) and assessment of glucose levels in interstitial fluid. All tests were carried out fasted in the morning hours (from 7:30 to 10:00 a.m.). Subjects consumed 150 g of boiled buckwheat groats in combination with 30 g inulin or 150 g of boiled buckwheat groats with 30 g of guar gum (Optifiber^®^) in accordance with the protocol of the study. Dietary fiber was dissolved in 200 mL of water and taken before eating porridge. Lactulose 20 g was used as a stand-alone test that does not require additional food intake, as is customary in the standard side test, to determine the characteristics of the microbiota composition. The study design and time frame of the study are presented in Figure 1.

### 2.3. Intervention

Inulin (Grafti@GR, standard inulin) was produced by BENEO-Orafti S.A. (3300, Tienen, Belgium) (composition: per 100 g—94 g inulin, 0 g protein, 0 g fat, and 6 g carbohydrates). Guar gum (Optifiber^®^), extract of Cyamopsis tetraganoloba fruit, was produced by Nestle Health Science (Deutschland) GmbH (67574, Osthofen, Germany). Lactulose 667 mg/mL (Portalac^®^ syrup) was produced by Belupo (48000, Koprivnika, Republic of Croatia).

### 2.4. Anthropometric and Metabolic Assessments

Height and body weight were measured once before the start of the study. BMI was calculated by dividing the weight (in kilograms) by the height (in meters squared).

#### 2.4.1. HbA1c Assessments

HbA1c was determined by the reference method (DCCT) of high-performance liquid chromatography (HPLC); the method is certified by NGSP. The analytical system used was Roche Cobas c8000 (Roche Diagnostics, Switzerland No. 2012/122722264 dated 19 September 2016).

#### 2.4.2. Glucose Detection

For continuous glucose monitoring (CGM), each participant was fitted with a FreeStyle Libre (FSL) sensor at least 2 days before the food load tests. Patients were trained in the use of the FSL sensor. For adequate data collection, it was recommended to read the sensor using a reader or a smartphone with the FreeStyle Libre Link app installed at least every 8 h, even outside of the tests. Glucose results were analyzed via the LibreView cloud-based monitoring system based on generated reports from compatible FreeStyle glucose monitoring devices. Determination of gases in exhaled air was performed during the hydrogen–methane breath test, a modern non-invasive method for diagnosing gut microflora disorders based on the determination of H_2_ and CH_4_ concentrations in exhaled air.

#### 2.4.3. Exhaled Gases Detection

The test consisted of collecting exhaled air samples (on an empty stomach and every 20 min after a food load for 3 h: 10 samples per breath test) in aluminized plastic bags (Guangzhou Itingbaby Tech Co. (Huangge Town, Nansha District, Guangzhou City, China) from which the air was analyzed for H_2_, CH_4_, and oxygen (O_2_) on a gas analyzer (GastroCH4ECK by Bedfont (Harrietsham, Maidstone Kent, ME17 1JA, England)) no later than 7 days after the breath test and exhaled air sampling. H_2_ and CH_4_ concentrations were adjusted for true oxygen content for standardization to alveolar gas levels and presented in ppm (parts per million). Subjects who had a peak CH_4_ production in exhaled air of 3 ppm or more during the 90 min test were PCH_4_. If a maximum of 20 ppm or more of H_2_ was produced in exhaled air during the 90 min test, the participant was classified as PH_2_. When concentrations of both gases exceeded the cut-off levels (CH_4_ ≥ 3 ppm and H_2_ ≥ 20 ppm), subjects were considered PCH_4_ + H_2_. Subjects who had low gas concentrations (CH_4_ < 3 ppm and H2 < 20 ppm) during the test were considered weak CH_4_ and H_2_ producers (WPCH_4_ + H_2_) [35].

### 2.5. Statistical Analysis

Statistical analysis of the results was performed using IBM SPSS Statistics software, version 26 using standard methods of variation statistics. All data are presented as median [quartile1; quartile3] (Me [Q_1_; Q_3_]) and mean ± standard deviation of the mean (Mean ± SD). Normality of distribution was checked using the Shapiro–Wilk criterion. Based on the amplitude of the glucose level A(G) from baseline and duration of its rise after food load, the relative area under the glycemic curve from 0 to 180 min (AUCrel(G)) was calculated. The amplitude of gases and tmax were analyzed after a food load. The area under the gas concentration curve from 0 to 180 min (AUCabs(H_2_) and AUCabs(CH_4_)) was calculated by trapezoid method. The A(H_2_) and A(CH_4_) was estimated as the difference between baseline (before DF intake) and the maximum level of the index. Analysis of variance with the Friedman test was used to compare data for dependent samples with non-normal distribution. The Mann–Whitney test was used for intergroup comparison of two independent samples. Nonparametric correlations were calculated using the Spearman rank correlation coefficient; parametric correlations were calculated using the Pearson correlation coefficient. Differences were considered statistically significant at *p* < 0.05. Since the study was a pilot project, the sample size was not calculated in advance.

## 3. Results

### 3.1. Subject Characteristics

The study included 14 patients with T2DM (11 female) and 14 controls (13 female). There were 83 food load tests: 41 tests in T2DM patients and 42 tests in the control group, including 27 with lactulose, 28 with guar gum, and 28 with inulin. One participant with T2DM did not have a lactulose test. HbA1c was not detected in the control group. The normoglycemic status was confirmed by daily glucose monitoring reports from Freestyle Libre. The median of the glucose management indicator (GMI) was 5.6% [5.2; 5.8]. The GMI provides an average estimate of the blood glucose levels over a specified period, typically the past 90 days. The characteristics of the study subjects are presented in Table 1.

The doses and type of glucose-lowering medication therapy in patients with T2DM did not change during the study. The majority of patients received metformin (71.4%), SU was prescribed in 57.1% of patients, SGLT-2 inhibitors in 50.0% of patients, DPP-4 inhibitors in 21.4%, and insulin in 14.3%. Statins were received by 42.9% of patients with T2DM. Subjects in the control group did not receive any medications.

### 3.2. Gas Production Analysis

Intestinal gas production hardly increased up to 90 min after lactulose administration in six patients with T2DM, while all those in the control group had gas production within 90 min. All subjects were split into hydrogen producers (PH_2_), CH_4_ and H_2_ producers (PCH_4_ + H_2_), or methane producers (PCH_4_) depending on their intestinal gas production before 90 min. The distribution of study subjects according to the release of H_2_ and CH_4_ is presented in Table 2.

Within 180 min after the lactulose administration, H_2_ production was observed in eleven patients with T2DM, including nine who were PH_2_, and in all control subjects, including eight who were PCH_4_ + H_2_(Table 2).

H_2_ production was higher in patients with T2DM after inulin loading and after the lactulose test in the control group (Table 3). The absolute area under the curve (AUCabs) of H_2_ and the amplitude (A) of H_2_ were significantly higher after inulin intake and lactulose test compared to guar gum intake (*p* = 0.032; *p* = 0.005), without significant differences between inulin intake and lactulose test.

AUCabs(H_2_) was significantly lower in patients with T2DM compared with the control group in the lactulose test (*p* = 0.043). The time period to reach maximum H_2_ levels (tmax(H_2_)) after lactulose administration was more in patients with T2DM—160 min [140; 180] compared with the control group—120 min [85; 140] (*p* = 0.022). There was no significant difference in tmax (H_2_) between the tests within each of the study groups.

The degree of CH_4_ increase (measured by AUCabs(CH_4_), A(CH_4_), and tmax (CH_4_)) did not differ significantly in the T2DM group compared to the control group. All types of nutritional loads had a tendency to increase CH_4_ elevation in the control group (Table 3).

### 3.3. Glucose Level Analysis

The glucose curves were analyzed in both groups. The amplitude (A(G)) and duration of the glucose rise were measured, and the relative area under the glycemic curve(AUCrel(G)) was calculated(Table 4).

Among all the loading tests, the minimal rise in glucose from baseline and AUCrel(G) was observed after the lactulose test, both in the group of T2DM patients and in the control group; the difference with the intake of DF was significant (Table 3). There was no significant difference in A(G) after inulin intake with buckwheat and after guar gum intake with buckwheat, as well as in AUCrel(G) between different types of DF added to buckwheat in each of the study groups (*p* > 0.05).

**Figure 2:**
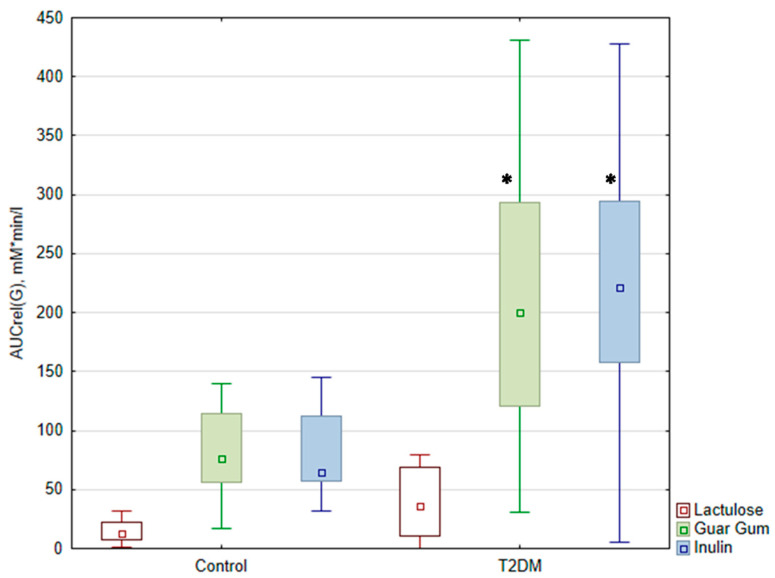
Relative area under the glycemic curve (AUCrel(G) (mM*min*L from 0 to 180 min)) during the test. The data are presented as medians with an interquartile range *—*p* < 0.05 compared with control. AUCrel(G)—the relative area under the glycemic curve from 0 to 180 min.

There was no significant difference in the A(G) and AUCrel(G) after lactulose administration between the patient group and the control group (*p* > 0.05). Upon the ingestion of buckwheat with DF, the expected increase in glycemic levels was more significant in patients with T2DM than in the control group (*p* < 0.05). The AUCrel(G) was significantly higher after taking buckwheat with DF in the group of patients with T2DM compared to the control group (Figure 2).

There was no significant difference in the duration of the glucose rise depending on the food load in both groups of subjects (*p* > 0.05). However, there was a tendency for a shorter duration of the glucose rise after taking buckwheat with DF in the control group compared to the group of patients with T2DM.

### 3.4. Correlation Analysis

**Figure 3:**
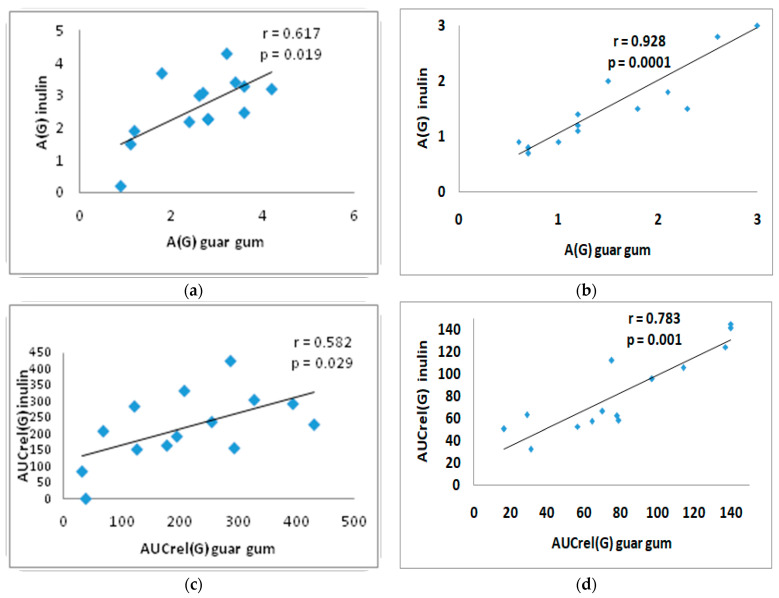
Correlation between A(G) after inulin intake and after guar gum intake in the T2DM group (**a**) and in the control group (**b**); and correlation between AUCrel(G) with inulin and with guar gum in the T2DM group (**c**) and in the control group (**d**). A(G)—amplitude of glucose level, AUCrel(G)—relative area under the glycemic curve from 0 to 180 min.

There was a direct significant correlation between A(G) with inulin and A(G) with guar gum: in the T2DM group (r = 0.617; *p* = 0.019) and in the control group (r = 0.928; *p* = 0.0001) (Figure 3a,b). A direct correlation was observed between AUCrel(G) with inulin and with guar gum: in the T2DM group (r = 0.582; *p* = 0.029) and in the control group (r = 0.783; *p* = 0.001) (Figure 3c,d).

**Figure 4:**
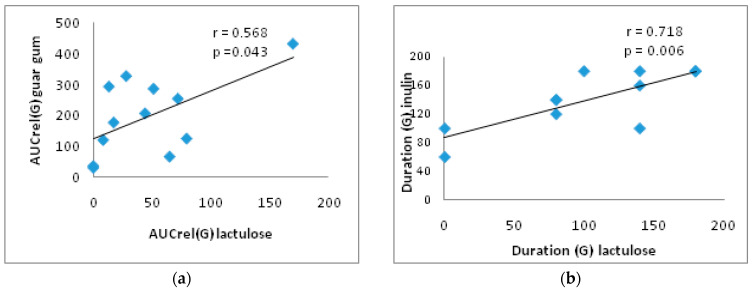
Correlation between AUCrel(G) with lactulose and with guar gum (**a**) and between duration (G) with lactulose and with inulin (**b**) in the T2DM group. AUCrel(G)—the relative area under the glycemic curve from 0 to 180 min.

In addition, in the group of T2DM patients, there is a direct significant correlation between the AUCrel(G) with lactulose and with guar gum (r = 0.568; *p* = 0.043) and between the duration of the glucose rise with inulin and with lactulose (r = 0.718; *p* = 0.006) (Figure 4).

**Figure 5:**
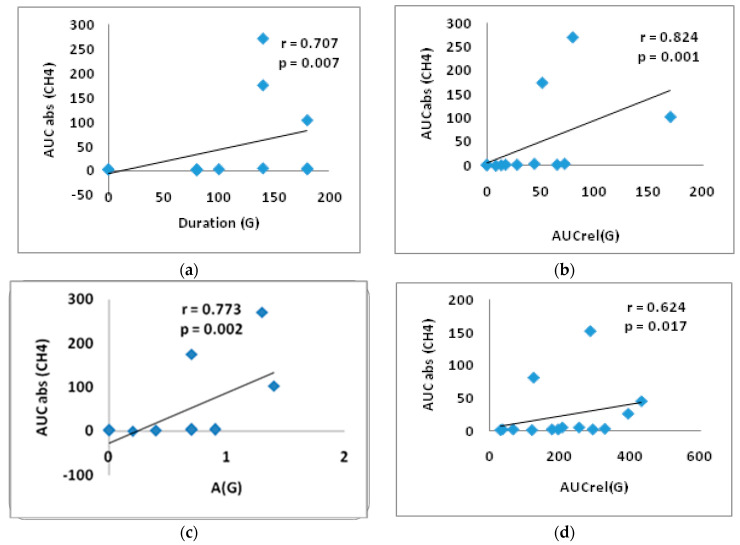
Correlation between AUCabs (CH_4_) and duration(G) (**a**); AUCabs (CH_4_) and AUCrel(G) (**b**); AUCabs(CH_4_) and A(G) in the lactulose test (**c**); AUCrel(G) and AUCabs (CH_4_) and in the test with guar gum in the T2DM group (**d**). AUCabs—absolute area under the gas concentration curve from 0 to 180 min, A(G)—the amplitude of glucose level, AUCrel(G)—the relative area under the glycemic curve from 0 to 180 min.

In the group of T2DM patients in the lactulose test, significant direct correlations were obtained between duration (G) and AUCabs(CH_4_) (r = 0.707, *p* = 0.007), between AUCrel(G) and AUCabs(CH_4_) (r = 0.824, *p* = 0.001), as well as between A(G) and AUCabs(CH_4_) (r = 0.773, *p* = 0.002) (Figure 5a–c). A significant direct correlation was identified between AUCrel(G) and AUCabs(CH_4_) (r = 0.624, *p* = 0.017) in the guar gum test, suggesting a direct association between glucose elevation and CH_4_ production (Figure 5d).

**Figure 6:**
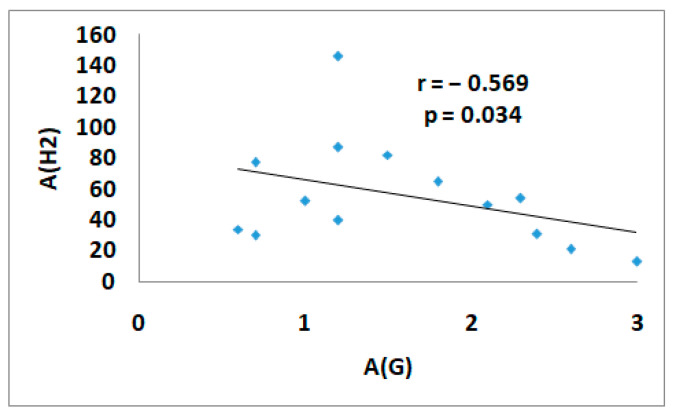
The correlation between A(G) and A(H_2_) in the test with guar gum in the control group. A(G)—the amplitude of glucose level, A (H_2_)—the amplitude of H_2_ level.

A significant inverse correlation between A(G) and A(H_2_) (r = −0.569, *p* = 0.034) was found after the guar gum intake in the control group. The maximum hydrogen rise was associated with a minimum rise in glucose levels (Figure 6).

## 4. Discussion

During our study, we obtained data on the changes in CH_4_ and H_2_ levels in exhaled air on the intake of different prebiotics and with food load in patients with T2DM, and we also analyzed correlations between individual indicators and postprandial glucose levels. The lactulose breath test showed that all control group subjects and only seven patients with T2DM had a rise in H_2_ production, with one patient showing isolated CH_4_ production and another not producing either CH_4_ or H_2_. An increase in hydrogen concentrations of more than 20 ppm from baseline within 90 min is recommended to be diagnostic of small intestinal bacterial overgrowth (SIBO) [19]. In our study, 5 T2DM patients of 14 and 11 from 14 in the control group could be diagnosed with SIBO. Our data on a small group of patients do not confirm the statement that the risk of SIBO in diabetic patients is 2.91 times higher than that in patients without diabetes [36]. Patients with T2DM produced lower levels of H_2_ and the peak of the maximal concentration of H_2_ was much later compared with the normal group. The appearance or the maximum in the expiratory breath of hydrogen (H_2_) produced by colonic fermentation of an ingested lactulose may reflect the orocecal transit time (OCTT) [37]. A delayed breath test has been reported in various sub-groups of patients, including those with T2DM [32,38].

People can be classified into different groups: those who release only H_2_ in exhaled air (indicating the absence of MetArchs), those who predominantly release CH_4_ (indicating the presence of many MetArchs consuming H_2_), those who release both CH_4_ and H_2_ (with relatively few MetArchs present), and those who do not release significant amounts of H_2_ and CH_4_ (indicating poor intestinal flora) [24,39,40,41]. Our study showed that the control group was characterized by the eubiosis and predominance of PCH_4_+/H_2_+. The presence of PCH_4−_/H_2_ in the group of diabetic patients confirms the presence of dysbiosis in diabetes mellitus. According to published experimental and clinical studies, the gut microbiome in diabetes is associated with an increase in MetArch abundance and a decrease in hydrogen-producing bacteria like Bifidobacterium, Bacteroides, Faecalibacterium, Akkermansia, and Roseburia [42]. MetArch produces CH_4_ by consuming H_2_, which decreases its concentration in the intestinal lumen and in the blood and decreases H_2_ components’ antioxidant activity. This can lead to obesity by increasing oxidative stress [43].

In our study, there was a decrease in H_2_ production in patients with T2DM with any food load compared to the control group, which could also indicate a decrease in the bacteria that produce hydrogen. Increased CH_4_ production is indicative of metabolic disorders and oxidative stress [44,45], while H_2_ synthesis is indicative of metabolic health and the state of antioxidant defense. Hydrogen is a product of the intestinal microflora’s fermentation of lactulose and dietary fiber. H_2_ can easily penetrate membranes and have antioxidant, anti-inflammatory, and anti-apoptotic effects [46]. The guar gum test showed an inverse correlation between A(G) and A(H_2_) in individuals with normoglycemia (r = −0.569, *p* = 0.034), with the most pronounced increase in H_2_ being associated with a smaller increase in glucose. Results confirm the maintenance of the level of enzymatic activity of the intestinal microbiota in control group subjects.

Inulin and guar gum intake have a similar effect on glycemic response, as demonstrated by the direct correlations between A (G) and AUCrel(G) in response to DF intake in our study [47]. According to our study, the type of DF can have varying effects on the production of H_2_. The production of H_2_ in patients with T2DM was higher after eating buckwheat porridge with inulin than after eating porridge with guar gum. We suppose that this results from the different chemical composition of the food fibers and the enzymatic activity of the microbiota involved [32,47,48].

After lactulose intake, in T2DM patients the peak of the H_2_ increase in the exhaled air took place at 160 min, whereas it was earlier (at 120 min) in the control group (*p* < 0.05). However, there was a tendency for higher tmax H_2_ levels after inulin intake. These changes may be related to the slow transit of food through the intestine in patients with T2DM [49,50,51] and the pharmacological properties of inulin [32]. When taking guar gum, the tmax H_2_ in patients with T2DM was not significantly lower than the control group subjects. The possible reasons for this may be due to the degree of the viscosity of the dietary fiber [32]. Another explanation for this fact has been revealed in the results of a study conducted by Paudel D. et al. in 2024, which suggests that guar gum may have an impact on intestinal conditions [52]. These findings require further research with a large number of subjects.

In contrast to the inverse correlation between H_2_ production and glucose levels in the control group, patients with T2DM in the lactulose test obtained reliable (*p* < 0.05) direct correlations between A(G) and AUCabs(CH_4_) (r = 0.773; *p* = 0.002); between A(G) and A(CH_4_) (r = 0.707; *p* = 0.007); and between AUCrel(G) and A(CH_4_) (r = 0.824; *p* = 0.001). The gut microbiota, particularly archaea, produces methane as a metabolite. The level of CH_4_ in exhaled air is a direct indicator of metabolic disorders in the body, as well as the severity of SIBO, which worsens the prognosis of cardiovascular diseases [53]. In the literature, there is evidence of the positive physiological properties of methane based on experimental data [54,55,56,57]. The trend observed in our study of higher CH_4_ production in the control group may be related to the greater production of H_2_, which is recycled by the methane-producing gut microflora during methane production [58].

Women were equally represented in the control and diabetes groups in our study. By excluding patients with gastrointestinal tract diseases from the study, we reduced the impact of these factors on the study results. According to the results of published studies, the influence of gender on the results of a breath test is contradictory. Some studies have confirmed the fact that women are the main methane producers [59]. However, in other studies, there were no differences in breath test results between men and women [60,61,62,63]. At the same time, Newberry C. et al. [64] found that with aging women have an increased likelihood of developing SIBO, which, as a consequence, leads to an increase in methane production compared to men. In another study, it was found that age has no impact on the results of the breath test in women [65].

According to the literature, the results of the relationship between the breath test and weight are contradictory. In studies involving adult patients, a correlation was found between increased weight and decreased hydrogen production or increased methane production [66,67]. However, other studies on children have shown a different trend: a positive breath test is linked to a lower body mass index (BMI) and a height-for-age Z-score, which indicates SIBO [60,68]. In 2020, two studies were published. One study did not show any effect of body mass index (BMI) on the breath test [65]. The other study found a weak relationship between BMI and increased gas production in women [59].

## 5. Study Limitations

The median age of the control group was slightly lower than that of the T2DM patients. The T2DM patients were obese and were on different variants of glucose-lowering medication, which may have influenced the composition of the intestinal microbiota and intestinal gas production.

In this study we used single administrations of inulin, guar gum, and lactulose to register the production of the gaseous biomarkers of metabolic gut microbiome activity. Differences in the responses of biomarker levels reflect differences in the taxonomic composition of the microbiome in investigated humans, but we could not confirm this due to the absence of the sequencing of the gut microbiota microorganisms. We have plans to follow this study with a long-term administration of the different prebiotics and a following analysis of the taxonomic composition of the microbiota.

Since this was a feasibility pilot study with only 28 participants, it is important to interpret our data with caution. To confirm our findings and provide more generalizable conclusions, it is necessary to conduct larger, more statistically powerful studies.

## 6. Conclusions

To our knowledge, this is the first study to investigate the relationship between postprandial glycemic levels based on flash-monitoring and the production of intestinal H_2_ and CH_4_ gases based on measurement of the amount of these gases in exhaled air. All subjects in the control group had a significant increase in exhaled H_2_. The level of H_2_ in exhaled air after food load in patients with T2DM was lower than in normoglycemic subjects. In normoglycemic subjects there was an inverse correlation between maximum glucose rise and maximum H_2_ in exhaled air after guar gum intake. At the same time, in patients with T2DM the level of CH_4_ in exhaled air directly correlated with the parameters of postprandial glycemia in the test with lactulose. The obtained data may indicate the existence of a relationship between the level of intestinal gases and postprandial glycemia. The further study of the relationship between intestinal gas production and postprandial glycemia in patients with T2DM in a larger sample and with different types of sugar-lowering therapy is needed. The confirmation of a causal relationship between decreased H_2_ production, increased intestinal CH_4_ production, and more severe postprandial glycemia may identify new therapeutic targets for the correction of postprandial glycemia in patients with T2DM.

## Figures and Tables

**Table 1:** Characteristics of study subjects.

Participant Characteristics	T2DM	Control
Number of subjects, (f ^1^)	14 (11)	14(13)
Age (years)	58 [54; 62]	46 (28; 56)
Weight (kg)	111.5 [92.0; 123.3]	63.5 (57.2; 68.2)
BMI ^2^ (kg/m^2^)	42.3 [35.6; 47.7]	22.6 (20.6; 25.8)
HbA1c ^3^ (%)	8.4 [6.9; 9.9]	-

Data are presented as Me [Q1; Q3]. ^1^ f—number of women from the total number of subjects in the group; ^2^ BMI—body mass index; ^3^ HbA1c—glycated hemoglobin.

**Table 2:** The distribution of the study subjects depending on the release of H_2_ and CH_4_ during a lactulose breath test.

Subject Characteristics	T2DM(n)	Control(n)
	up to 90 min of the test	
PCH_4_+/H_2_+	1	7
PCH_4_+	2	1
PH_2_+	4	4
PCH_4_−/H_2_−	6	2
	up to 180 min of the test	
PCH_4_+/H_2_+	2	8
PCH_4_+	1	0
PH_2_+	9	6
PCH_4_−/H_2_−	1	0

PCH_4_+—methane release ≥ 3 ppm; PH_2_+ hydrogen release ≥ 20 ppm; PCH_4_+/H_2_+ simultaneous release of methane ≥ 3 ppm and hydrogen ≥ 20 ppm; and PCH_4_−/H_2_−—methane release < 3 ppm, hydrogen < 20 ppm.

**Table 3:** Indicators of gas production during tests with lactulose and dietary fibers in patients with T2DM and control group.

Groups	Me (1)[Q1; Q3] (2) Mean ± SD	Lactulose		Guar Gum		Inulin	
		H_2_	CH_4_	H_2_	CH_4_	H_2_	CH_4_
**T2DM**	**AUCabs** (ppm*h)	(1) 96 [65; 118] (*2*)*93 ± 41*	(1) 2 [1; 4] (*2*) *44 ± 87*	(1) 43 [34; 70] (*2*) *50 ± 28*	(1) 3 [2; 21] (*2*) *23 ± 44*	(1) 105 [70; 151] (*2*) *117 ± 62*	(1) 4 [1; 44] (*2*) *38 ± 69*
	**A** (**ppm**)	(1) 61 [30; 66] (*2*) *58 ± 36*	(1) 1 [1; 2] (*2*) *11 ± 20*	(1) 17 [8; 33] (*2*) *23 ± 19*	(1) 1 [−1; 2] (*2*)*2 ± 5*	(1) 71 [44; 81] (*2*) *69 ± 31*	(1) 1 [−1; 2] (*2*)*2 ± 5*
	**tmax** (**min**)	(1) 160 [140; 180] (*2*) *149 ± 36*	(1) 60 [20; 140] (*2*) *75 ± 65*	(1) 140 [80; 160] (*2*) *120 ± 60*	(1) 30 [0; 75] (*2*) *41 ± 45*	(1) 170 [120; 180] (*2*) *148 ± 37*	(1) 40 [0; 110] (*2*) *54 ± 58*
**Control group**	**AUCabs** (ppm*h)	(1) 126 (98; 203) * (*2*) *148 ± 68*	(1) 5 (1; 25) (*2*) *62 ± 111*	(1) 51 (32; 74) (*2*) *56 ± 34*	(1) 14 (1; 41) (*2*) *52 ± 89*	(1) 123 (86; 212) (*2*) *140 ± 80*	(1) 8 (1; 30) (*2*) *65 ± 118*
	**A** (**ppm**)	(1) 75 (60; 111) (*2*) *81 ± 35*	(1) 2 (0; 4) (*2*) *20 ± 41*	(1) 16 (12; 26) (*2*)*20 ± 15*	(1) 2 (1; 6) (*2*)*9 ± 17*	(1) 74 (41; 84) (*2*)*68 ± 37*	(1) 1 (−1; 9) (*2*)*9 ± 22*
	**tmax** (**min**)	(1) 120 (85; 140) * (*2*) *116 ± 38*	(1) 30 (5; 60) (*2*)*49 ± 56*	(1) 160 (125; 160) (*2*) *146 ± 35*	(1) 70 (20; 115) (*2*)*70 ± 54*	(1) 120 (100; 155) (*2*) *116 ± 50*	(1) 50 (0; 75) (*2*) *53 ± 52*

Data are presented as (1) Me [Q1; Q3] and (2) *Mean ± SD*. * *p* < 0.05—between T2DM and control group; AUCabs—absolute area under the gas concentration curve from 0 to 180 min; A—amplitude; and tmax—time to reach maximum level.

**Table 4:** Glucose levels during tests in T2DM patients and control group.

(1) Me [Q1; Q3] (2) Mean ± SD	Lactulose (a)		Guar Gum (b)		Inulin (c)	
	T2DM	Control	T2DM	Control	T2DM	Control
**A** (**G**) (**mM/L**)	(1) 0.7 [0.3; 0.9] (*2*) *0.7 ± 0.5*	(1) 0.3 (0.2; 0.5) (*2*) *0.6 ± 0.7*	(1) 2.8 ^†^ [1.7; 3.5] (*2*) *2.6 ± 1.02*	(1) 1.4 (1.1; 2.2) * (*2*) *1.6 ± 0.8*	(1) 2.8 [2.1; 3.3] ^†^ (*2*) *2.6 ± 1.0*	(1) 1.5 (1.0; 2.0) * (*2*) *1.6 ± 0.8*
**Duration** (**min**)	(1) 80 [120; 170] (*2*) *108 ± 63*	(1) 80 (80; 95) (*2*) *90 ± 44*	(1) 180 [140; 180] (*2*) *154 ± 37*	(1) 100 (100; 175) (*2*) *123 ± 44*	(1) 140 [115; 180] (*2*) *143 ± 37*	(1) 100 (85; 150) (*2*) *117.1 ± 41.4*
**AUCrel**(**G**) (mM*min/L)	(1) 36 [9.6; 70.3] (*2*) *45.7 ± 48.1*	(1) 13 (8.5; 21) (*2*) *30 ± 47*	(1) 200.7 ^†^ [107.4; 301.5] (*2*) *210.3 ± 126.9*	(1) 76.6 (58.4; 109.8) * (*2*) *81 ± 41*	(1) 221.6 ^†^ [157.1; 297.0] (*2*) *221.8 ± 107.6*	(1) 64.8 (57.3; 110.4) * (*2*) *83 ± 37*

The data are presented as (1) Me [Q1; Q3] and(*2*) *Mean ± SD*. A(G)—the amplitude of glucose level, AUCrel(G)—the relative area under the glycemic curve from 0 to 180 min, p—the validity of the differences; (a–b); [no differences with (b–c)]. * *p* < 0.01; ^†^ *p* < 0.0001.

## Data Availability

The original contributions presented in the study are included in the article, further inquiries can be directed to the corresponding author.
